# Inertial Indoor Pedestrian Navigation Based on Cascade Filtering Integrated INS/Map Information

**DOI:** 10.3390/s22228840

**Published:** 2022-11-15

**Authors:** Menghao Fan, Jia Li, Weibing Wang

**Affiliations:** 1 Institute of Microelectronics of Chinese Academy of Sciences, Beijing 100029, China; 2 School of Integrated Circuits, University of Chinese Academy of Sciences, Beijing 100049, China

**Keywords:** strapdown inertial navigation system, extended complementary filtering, particle filter, indoor positioning, map matching

## Abstract

Indoor pedestrian positioning has been widely used in many scenarios, such as fire rescue and indoor path planning. Compared with other technologies, inertial measurement unit (IMU)-based indoor positioning requires no additional equipment and has a lower cost. However, IMU-based indoor positioning has the problem of error accumulation, resulting in inaccurate positioning. Therefore, this paper proposes a cascade filtering algorithm to correct the accumulated error using only a small amount of map information. In the lower filter, the zero-velocity correction and the attitude-extended complementary filtering (ECF) algorithm are utilized to initially solve the pedestrian’s trajectory. In the upper filter, a particle filter (PF) combined with the map information is adopted to correct the accumulated error of the heading and stride length. In the 2D positioning process, the root mean square error (RMSE) of the proposed algorithm is only 1.35 m. In the altitude correction, this paper proposes a method of clustering floor discrimination to deal with the instability of the barometer resulting from an uneven pressure and temperature. In the final 3D positioning experiment, with a total length of 536.5 m and including the process of going up and down the stairs, the end-point error is only 2.45 m by the proposed algorithm.

## 1. Introduction

In recent years, location-based services (LBS) have been receiving extensive attention. Indoor positioning is very important in a wide variety of places, such as path planning in large buildings, airports, subway stations and other places, and positioning in emergency situations, such as fires and earthquakes [1]. Global Navigation Satellite Systems (GNSS), such as the Global Positioning System (GPS), can provide accurate and reliable positioning results but only in outdoor environments [2]. In an indoor environment, due to interference, such as signal attenuation and multipath effects, the performance of GNSS will drop sharply. At present, several positioning technologies suitable for different indoor environments have been developed.

Specifically, the indoor positioning technologies include Wi-Fi [3], UWB [4], ultrasonic [5], vision, etc. However, these technologies need to arrange some additional facilities in advance, which significantly increases the system cost. Moreover, the positioning precision of these technologies is significantly affected by the external interference. Meanwhile, other low-cost positioning technologies, such as Bluetooth technology [6] and radio frequency identification technology (RFID) [7], have a limited positioning accuracy and range. Compared with the above technologies, the inertial sensor-based indoor positioning technology is a low-cost positioning solution that does not require any additional facility and is not easily affected by the environment. However, the independent inertial navigation system (INS) faces the problem of error accumulation in practical use, and it needs to be integrated with other sensors or auxiliary information (Wi-Fi, Bluetooth, UWB, building structure information, etc.) to improve the positioning accuracy.

### 1.1. Related Work

Nguyen et al. [8] proposed a Range-Focused Function of Camera-IMU-UWB method to achieve the positioning with less drift. Zhu et al. [9] proposed a Wi-Fi/Bluetooth and pedestrian dead reckoning (PDR) fusion positioning method to solve the problem of the large accumulated error of the PDR. Wen et al. [10] used the UWB positioning technology to improve the positioning accuracy of the inertial navigation. However, these methods have a high installation cost and their maintenance and updates require much additional time and money. Some researchers corrected the inertial navigation error through Wi-Fi fingerprints [11,12], geomagnetic fingerprints [13,14], optical fingerprints [15] and other technologies. However, these methods require a lot of fingerprint collection work in advance, and the data need to be frequently maintained due to environmental interference.

The map-based IMU indoor positioning is a cost-effective method that requires no additional equipment, and the map information does not change with environmental factors. Shi et al. [16] proposed a PF-SLAM indoor pedestrian localization algorithm based on a feature-point map and corrected the inaccurate heading angle in the PDR positioning by turning the straight-state threshold detection. Koroglu et al. [17] proposed a non-recursive Bayesian map matching [18] approach to avoid the computational burden of the PF. Furthermore, Zhang et al. [19] proposed a method with a cascade filtering structure. The upper PF fused with the map information corrected the estimation result of the lower filter, thereby correcting the navigation error and improving the positioning accuracy.

Common solutions for the INS and auxiliary information integration, such as the Kalman filter, extended Kalman filter (EKF) and complementary filtering (CF), all have poor positioning accuracy on nonlinearly integrated navigation systems. To improve the accuracy of the INS, the nonlinear estimation using dynamic models that do not require linearization is a plausible solution. The PF is a completely nonlinear filter in the Bayesian framework, which can have a higher positioning accuracy estimation in highly nonlinear problems with arbitrary distributions. It has great flexibility in exploiting indoor building structure information [19]. However, using the PF alone has problems, such as a huge computational burden, easy failure and particle poverty [20].

Considering the advantages and disadvantages of both filters and aiming at the problem of error accumulation when the traditional filter is used alone, and the problem of a large amount of calculation when the PF is used alone, we adopt a cascade filtering algorithm [10,19].

### 1.2. Our Contributions

In this paper, the cascaded filtering method is used to combine the pedestrian motion characteristics obtained from the IMU with the indoor structure information to achieve an indoor location. This method only utilizes a small amount of map information to reduce the time and economic costs and requires no additional equipment and data maintenance. For the altitude correction, this paper proposes a floor discrimination method based on a cluster analysis to solve the problem of unstable barometer data caused by an uneven pressure and temperature. Finally, through the devices of an MAGR sensor ICM-2098, an air pressure sensor BMP280 and a low-power MCU STML103, this paper builds an autonomous pedestrian positioning system, which completes the indoor 3D positioning, including the complex process of pedestrians going up and down stairs, and obtains a good positioning performance.

## 2. Cascade Filtering Overall Framework

**Figure 1:**
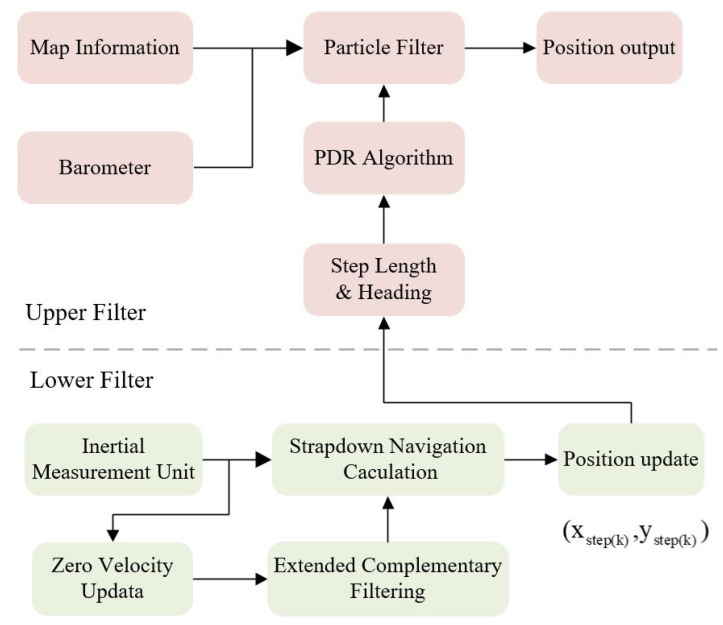
The overall frame diagram of the cascade filtering algorithm.

This research designs a cascade filtering algorithm using the information from microelectromechanical system (MEMS) sensors and indoor building structures. The lower filter preliminarily corrects the navigation accuracy through the ECF algorithm and the zero-velocity correction process, which provides a better inertial navigation solution for the upper PF, and solves the problem of the PF’s huge computation and lacking particles [21]. The iterative process of the upper PF uses the stride length and heading information obtained by the preliminary solution of the lower filter process. In addition, the upper filter combines the map information to eliminate the accumulated error of the heading angle caused by the large interference in the lower filter. By combining the barometer information to eliminate the drift in the height direction, we can realize the 3D navigation and positioning based on the building information, and the process is shown in Figure 1.

## 3. Lower Filter

Combined with the characteristics of pedestrian walking, the zero-velocity interval is obtained by the threshold method. In the zero-velocity range, we use the ECF algorithm to correct the attitude information of the sensor by correcting the accumulated error of the gyroscope. In the non-zero-velocity range, combined with the corrected attitude information, we project the acceleration vector into the navigation coordinate system. Then, the velocity and position information of the carrier are solved through the two integration processes of the acceleration, and the initial solution trajectory is provided for the upper filter.

### 3.1. Attitude Correction Based on ECF

In the long-term measurement, errors inevitably exist in the attitude calculation process due to the accumulated error of the gyroscope. The sensor’s attitude information is an important component in inertial navigation. It affects the acceleration projection in SINS, which in turn affects the accuracy of the trajectory restoration.

The complementary filtering is an important algorithm applied in the attitude and heading reference system (AHRS). This algorithm fuses the sensor data to estimate the attitude and has a higher computational efficiency than the Kalman filter [22]. The Mahony algorithm [20] is a classic algorithm based on nonlinear complementary filtering and has been widely used in low-cost inertial navigation. Based on the Mahony algorithm, reference [22] introduced the ECF algorithm. The algorithm was improved to solve the following problems in the attitude calculation process, such as a slow convergence and the failed decoupling with a pitch angle and roll angle in a heading angle calculation. The main idea of the ECF algorithm is introduced below.

When the attitude quaternion *q* and angular velocity *w* of the carrier are known, the iterative differential equation of the quaternion is given by:

(1) $$\dot{q}=\frac{1}{2}q⊗\left[\begin{array}{c} 0\\ w \end{array}\right]$$

Because the error of the gyroscope continues to accumulate over time, the error term *e* is used for correction, and the global parameter *k* is the gain used to scale the error correction term. Then, Equation (1) can be changed to:

(2) $$\dot{q}=\frac{1}{2}q⊗\left[\begin{array}{c} 0\\ w+ke \end{array}\right]$$

The error correction term *e* includes two parts: the accelerometer fusion correction term $e_{a}$ and the magnetometer fusion correction term $e_{m}$.

(3) $$\begin{array}{c} e_{a}=\frac{{}^{b}a}{∥{}^{b}a∥}\times \left(q_{k-1}^{*}⊗\left[\begin{array}{c} 0\\ {}^{n}v \end{array}\right]⊗q_{k-1}\right)\\ \;\;\;=\frac{{}^{b}a}{∥{}^{b}a∥}\times \left[\begin{array}{c} 2q_{1}^{2}q_{3}^{2}-2q_{0}^{2}q_{2}^{2}\;\\ 2q_{2}^{2}q_{3}^{2}+2q_{0}^{2}q_{1}^{2}\;\\ 2q_{0}^{2}+2q_{3}^{2}-1 \end{array}\right] \end{array}$$

where ${}^{n}v=\left[1,0,0\right]$ denotes the gravity vector in the navigation coordinate system, ${}^{b}a$ denotes the acceleration vector in the carrier coordinate system obtained from the measurement and the error term $e_{a}$ represents the total acceleration experienced by the pedestrian in the zero-velocity range which is consistent with the gravitational acceleration. Therefore, the gravity vector can be used as the correction reference for the attitude information.

(4) $$\begin{array}{c} e_{m}=\frac{{}^{b}m\times^{b}a}{∥{}^{b}m\times^{b}a∥}\times \left(q_{k-1}^{*}⊗\left[\begin{array}{c} 0\\ {}^{n}m{⊗}^{n}v \end{array}\right]⊗q_{k-1}\right)\\ \;\;\;\;\;=\frac{{}^{b}m\times^{b}a}{∥{}^{b}m\times^{b}a∥}\times \left[\begin{array}{c} 2q_{1}^{2}q_{2}^{2}+q_{0}^{2}q_{3}^{2}\;\\ 2q_{0}^{2}+2q_{2}^{2}-1\;\\ 2q_{2}^{2}q_{3}^{2}-2q_{0}^{2}q_{1}^{2} \end{array}\right] \end{array}$$

where ${}^{n}m=\left[m_{x},0,m_{z}\right]$ denotes the magnetic reference vector in the navigation coordinate system, and ${}^{b}m$ denotes the magnetic force vector in the carrier coordinate system obtained from the measurement.

Because the heading angle has no projection in the direction of the gravity vector, the error term ${}^{b}m$ is introduced. In order to eliminate the influence of magnetic tilt, the magnetic force vector and the gravity vector are subjected to cross multiply to obtain the vector pointing east. Then, the vector pointing east can be used as the correction reference for the heading angle. The complete error term e can be obtained by combing the two error terms:

(5) $$\begin{array}{c} e=\left\{\begin{array}{cc} e_{a}+e_{m} & \left(m_{\min}<∥{}^{b}m∥<{m_{\max}}_{}0<∥{}^{b}v∥\right)\;\\ e_{a} & \left(0<∥{}^{b}v∥\right)\;\\ {\left[0\;0\;0\right]}^{T} & else \end{array}\right. \end{array}$$

where

(6) $$\begin{array}{c} {}^{b}v=\left\{\begin{array}{cc} {\left[0\;0\;0\right]}^{T} & ∥\left(∥{}^{b}v∥-∥g∥\right)∥>g_{d}\;\\ {}^{b}v & else \end{array}\right. \end{array}$$

where $g_{d}$ is a small constant, *g* represents the gravitational acceleration and $\left[m_{min},m_{max}\right]$ represents the normal fluctuation range of the magnetic force vector norm. Equation (5) indicates that when the accelerometer’s measurement deviates from the gravitational acceleration by a large amount, or the magnetic vector deviates from the normal range, the corresponding part is removed from the total error term.

(7) $$\begin{array}{c} K=\left\{\begin{array}{cc} K_{norm} & if\;t>t_{init}\;\\ K_{norm}+\frac{t_{init}-t}{t_{init}}\left(K_{init}-K_{norm}\right) & else \end{array}\right. \end{array}$$

The gain *K* controls the response of the algorithm to change in the accelerometer and magnetometer. A larger value for *K* at the initial moment is defined as $K_{init}$, which can minimize the time required for the attitude angle to converge to the correct direction. After the initial process correction, the difference between the direction obtained from the gyroscope integration rotation and its actual direction is low. *K* should be relatively small at this time, and we can define it as $K_{norm}$. By adjusting the parameter *K*, the algorithm can achieve the best performance, then realize the real-time solution to the carrier [22].

### 3.2. Trajectory Restoration Process of Lower Filtering

After the space attitude of the carrier is corrected by the ECF algorithm, the carrier velocity and position can be further calculated as the output of the navigation system.

The rationale for updating the velocity and position based on Newton’s law of inertia. After collecting the current acceleration data in the non-zero-velocity range, the acceleration vector needs to be transformed in the coordinate system to remove the gravitational acceleration component from it, which is shown as Equation (8):

(8) $${}^{n}a={\hat{q}}_{k}{⊗}^{b}a_{k}⊗{\hat{q}}_{k}^{*}{-}^{n}g$$

where ${\hat{q}}_{k}$ represents the estimated attitude of the carrier corrected by the ECF algorithm, and ${}^{n}g$ represents the gravitational acceleration component. Then, through the integration process, the velocity and position of the carrier in the navigation system are updated as Equation (9):

(9) $$\begin{array}{c} \begin{array}{c} {}^{n}v_{k}{=}^{n}v_{k-1}+\frac{1}{2}{(}^{n}a_{k-1}{+}^{n}a_{k})\Delta t\\ {}^{n}p_{k}{=}^{n}p_{k-1}+\frac{1}{2}{(}^{n}v_{k-1}{+}^{n}v_{k})\Delta t \end{array} \end{array}$$

where Δ*t* is the sampling period, ${}^{n}v_{k}$ and ${}^{n}p_{k}$ represent the velocity and position finally calculated by the lower filter, respectively.

## 4. Upper Filter

In the indoor environment with a disordered magnetic field, the correction effect of only using the lower filter is limited. The accumulated error of the inertial sensor will make the pedestrian often have “go through the wall behavior” which is impossible in the actual walking process in the navigation process. So, we need to combine other auxiliary information to further correct the accumulated error. The particle filtering process combined with the map information can make full use of the indoor building structure information and use it as a measurement resource to constrain the trajectory of the pedestrians, correct the particle quality and improve the accuracy of the scheme.

Combined with the trajectory information $\left(x_{step_{k}},y_{step_{k}}\right)$ initially obtained by the lower-layer filtering, the stride and heading angle information $\left(S_{k},\theta_{k}\right)$ obtained by the inverse solution are used as the input information of the upper-layer particle filtering process. The indoor plane information can be used to constrain the pedestrian trajectories, reduce the computational burden of the PF and serve as the basis for a particle weight update. The height information of the building can be combined with the barometer data for a 3D trajectory reconstruction.

### 4.1. Map Modeling

**Figure 2:**
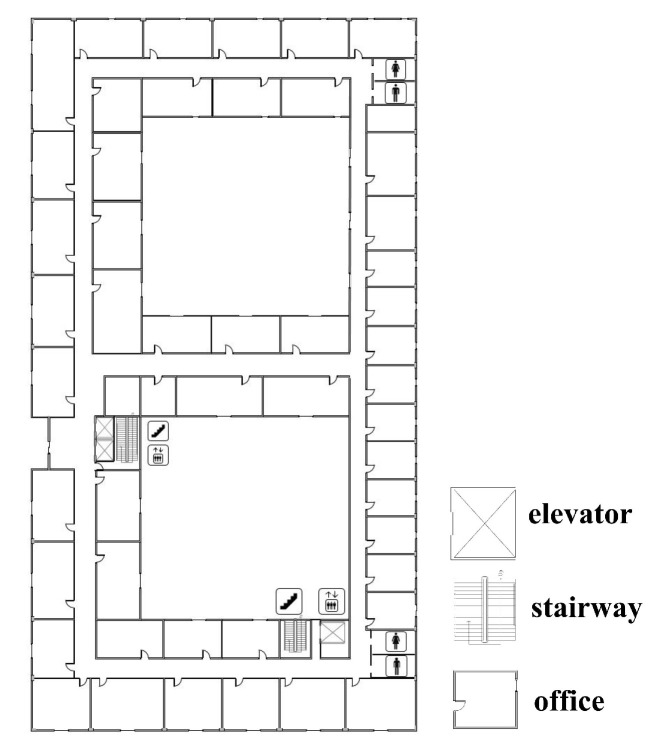
Construction information.

Without the need to install additional equipment, utilizing the map information based on building structures is a stable and cost-effective method for correcting an accumulated error. Most interior buildings are made up of corridors and rooms. We call the corridor paths the main indoor paths, which reflect the general situation of the interior of a building [16]. All the particles on the map can be repositioned according to the stride and heading obtained by the lower filter. During a particle propagation, map constraints are used to constrain the movement of particles. We create two different map constraints during the particle initialization and propagation, the PFXM and PFMK [23]. The PFXM indicates unconstrained mapping, where the particles can enter feasible and forbidden areas. In the PFMK, the particles are constrained to the main road and stairs of the building. The plane information is shown in Figure 2.

**Figure 3:**
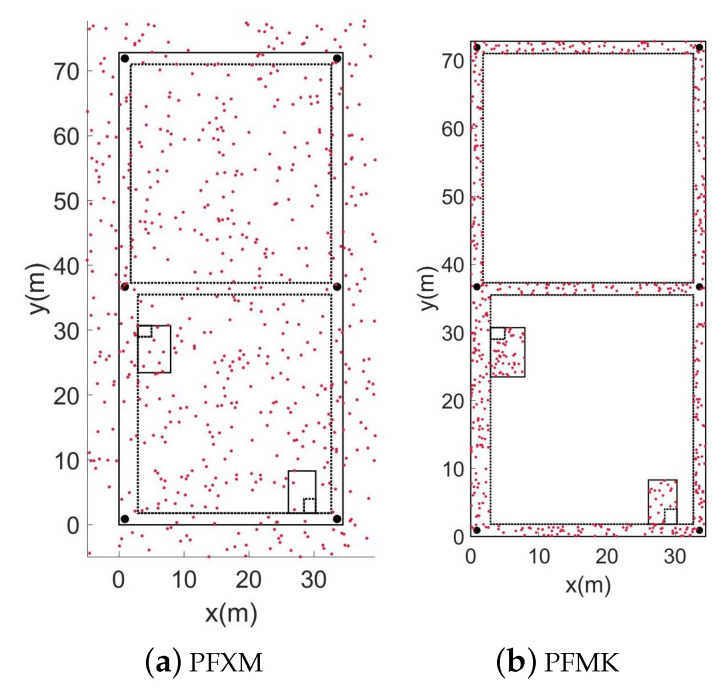
Feasible domain distribution of particles under different constraints.

The solid black line in Figure 3 represents the restricted range of the feasible area, and the dashed line represents the restricted range of the forbidden area. The black dot indicates the inflection point of the building’s map. During the iteration, particles entering the forbidden area will be invalidated. When there are too many failed particles, resampling is required.

### 4.2. Particle Filter Process Based on Map Matching

The PF is a recursive Bayesian filter using the Monte Carlo method. This filter represents the posterior probability density of random events through a set of random particle samples with weights in the state space and then estimates the state of the dynamic system from noisy or incomplete observation sequences. It can provide higher positioning accuracy in highly nonlinear problems with arbitrary distributions and has great flexibility in exploiting the indoor structural information of buildings [24].

#### 4.2.1. Particle Initialization

The initialization of particles follows a Gaussian distribution N(μ,δ).

(10) $$f\left(x\right)=\frac{1}{\sigma \sqrt{2\pi }}e^{-\frac{1}{2}\left(\frac{x-\mu }{\sigma }\right)}$$

#### 4.2.2. Particle Propagation

The state propagation equation for particle filter is:

(11) $$\left[\begin{array}{c} x_{k}\\ y_{k} \end{array}\right]=\left[\begin{array}{c} x_{k-1}+S_{k}\cos\theta_{\left(k\right)}\\ y_{k-1}+S_{k}\sin\theta_{\left(k\right)} \end{array}\right]+u_{k}$$

where $S_{k}$ is the stride length of the *k* th step and $\theta_{\left(t\right)}$ is the heading of the *k* th step. Both the two parameters are obtained by the lower filtering process. In addition, *u* is Gaussian noise with zero mean [25]. Both step size and heading can be expressed as:

$$S_{k}=\sqrt{{\left(x_{step(k)}-x_{step(k-1)}\right)}^{2}+{\left(y_{step(k)}-y_{step(k-1)}\right)}^{2}}$$

$$\theta_{\left(k\right)}=a\sin\left(\frac{y_{step(k)}-y_{step(k-1)}}{x_{step(k)}-x_{step(k-1)}}\right)$$

where $\left(x_{step(k)},y_{step(k)}\right)$ represents the position coordinates of the *k* th step calculated by the lower filter.

#### 4.2.3. Weight Update

After the particle propagation, if part of the newly generated particle swarm enters the forbidden area, this part of the particle is considered invalid, and its weight is set to zero. Otherwise, the particle weight is maintained. Near the inflection point of the building’s map, if the state of the pedestrian satisfies the condition of the position correction, the particle weight is updated according to Equation (12). Otherwise, the particle weight will be maintained.

(12) $$\begin{array}{c} w_{k}^{i}=\left\{\begin{array}{c} 0\;\;\;Particles\;through\;walls\;\\ \frac{1}{\sqrt{2\pi }\sigma }e\left[-\frac{\left(x^{i}\left(k\right)-x\left(k\right)\right)+{\left(y^{i}\left(k\right)-y\left(k\right)\right)}^{2}}{2\sigma^{2}}\right] \end{array}\right. \end{array}$$

#### 4.2.4. Particle Initialization

The weight of the particle at time *k* is normalized by:

(13) $$w_{x}^{i}\left(k\right)=w_{x}^{i}\left(k\right)/\sum_{i=1}^{N}w_{x}^{i}\left(k\right)$$

#### 4.2.5. Resampling

Resampling the normalized weighted samples, the formulas for resampling are shown as Equation (15):

(14) $$N_{eff}<\frac{1}{2}N$$

(15) $$N_{eff}=\frac{1}{\sum_{i=1}^{N}{\left(W_{t}^{i}\right)}^{2}}$$

When the effective particle number $N_{eff}$ is lower than the threshold, the roulette method is used for systematic resampling to retain the particles with large weights as much as possible. After the particle swarm is normalized, the higher the weight of the particle, the larger the area in the roulette wheel, and the greater the probability of being drawn. Therefore, the system resampling by the roulette method is used to keep particles with a high weight as much as possible. Then, if $\sum_{i=1}^{N}\bar{w_{x}^{i}}\left(k\right)\geq u$, the particles with the corresponding weight are copied, where *u* represents random in 0 to 1 [23].

### 4.3. Pedestrian State Determination

The purpose of the pedestrian state determination is to determine whether the pedestrian is in the two states of straight walking or single turning. In order to correct the heading angle in the straight walking state, correct the position information at the inflection point of the building’s map. The specific process is shown in Algorithm 1.

The drift angle $\theta_{\Delta k}$ is the difference between the heading angles $\theta_{\left(k\right)}$ at two adjacent moments provided by the lower filter when the pedestrian is stationary:

(16) $$\theta_{\Delta k}=\theta_{\left(k\right)}-\theta_{\left(k-1\right)}$$

In order to better judge the pedestrian state, we define the sum of n drift angles before and after the *k* th step as:

(17) $${\hat{\theta }}_{\Delta k}=\sum_{i=k-n}^{k+n}\theta_{\Delta i}$$

We also define the mean of the sum of *n* drift angles before and after the *k*th step as ${\bar{\theta }}_{\Delta k}$ and the standard deviation as $\sigma_{\Delta k}$:

(18) $${\bar{\theta }}_{\Delta k}=\sum_{i=k-n}^{k+n}\frac{{\hat{\theta }}_{\Delta i}}{2n+1}$$

(19) $$\sigma_{\Delta k}=\sqrt{\frac{\sum_{i=k-n}^{k+n}\left({\hat{\theta }}_{\Delta i}-{\bar{\theta }}_{\Delta k}\right)}{2n+1}}$$

**Algorithm 1:**Pedestrian walking state determination algorithm**Input:** Heading angle: θ. Threshold: val**Output:** Whether the pedestrian is in a straight walking state or in a single turning state1:set $\eta_{k}=\left[\theta_{\Delta (k-n)}^{},\theta_{\Delta (k-n+1)}^{},\cdots \theta_{\Delta (k+n-1)}^{},\theta_{\Delta (k+n)}^{}\right]$2:**if** $std(\eta_{k})\leq val$ **then**3:   %Indicates that the *n* heading angles before and after the *k*th step are basically the same4:**return** Pedestrian’s walking state in a straight line5:**else**6:   **define** ${\hat{\theta }}_{\Delta k}=\sum_{i=k-n}^{k+n}\left(\theta_{\Delta i}\right)$7:        According to Equations (18) and (19), the mean and variance of ${\hat{\theta }}_{\Delta k}$ can be expressed as ${\bar{\theta }}_{\Delta k}$ and $\sigma_{\Delta k}$8:      **if** $\sigma_{\Delta k}\leq val$ && ${\bar{\theta }}_{\Delta k}>val$ **then**9:     %Indicates that there is a sudden increase in the drift angle near the *k*th step, but the *n* drift angles before and after it are small10: **return** Pedestrian’s walking state in a single turn11:   **else**12:     **return** None13:   **end if**14:**end if**

### 4.4. Correction Process Combined with Map Information

When a pedestrian trajectory hits the wall, it means that there are too many failed particles in the particle swarm and resampling is required.

If the pedestrian is walking in a straight line at this point and is in the main straight corridor of the building, then the heading drift caused by the accumulated error of the gyroscope can be corrected according to the map information. Otherwise, a simple resampling process is performed without heading correction.

Near the inflection point of the building’s map, if the pedestrian is in a single-turn state at this time, the weight of the particle can be assigned according to the map information, and then the deviated particle group can be corrected to the corresponding inflection point. In this way, the drift in the mileage caused by the accumulated error of acceleration can be corrected. The position correction process of the particle swarm is as follows:

1. According to Algorithm 1, determine whether the pedestrian is in a single-turn state and is close to the inflection point of the building’s map. If so, go to the next step.
2. Update the particle weights according to Equation (12), and then perform particle resampling.
3. Perform clustering analysis on the resampled particle, and then the cluster center with the highest weight can be determined as the final corrected position.

**Figure 4:**
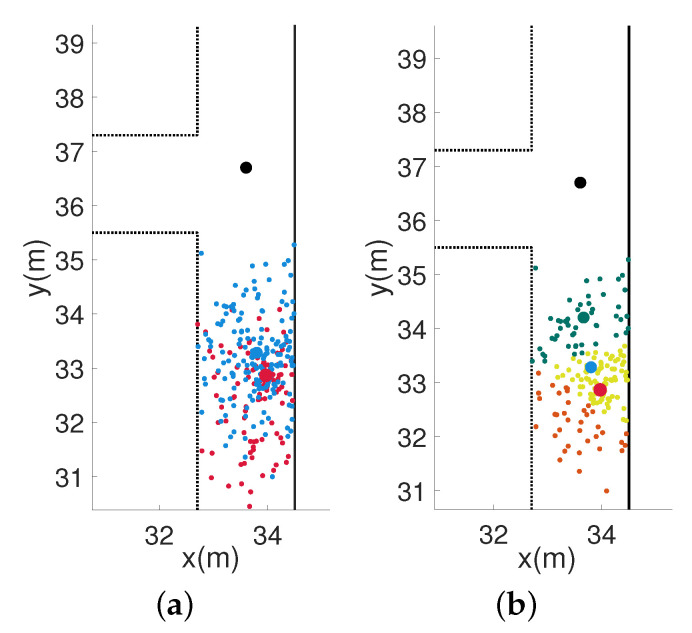
Corrected position information near the inflection point of the map. (**a**) Before position correction. (**b**) After position correction.

In Figure 4a, the red scatter points represent the original particle swarm, and the blue scatter points represent the resampled particle swarm. Figure 4b shows the results of the clustering analysis according to the weight on the resampled particle swarm. From Figure 4b, we can see that the position of the center point of the cluster with the highest weight is determined as the corrected position, which is the center position of the green particle cluster in the figure. The black dot is the position of the inflection point of the building’s map.

## 5. Experimental Environment and Display

### 5.1. Hardware

The main function of the hardware part is to collect inertial, magnetic, air pressure and temperature data of foot movement through the MARG sensor and barometer. After the preliminary protocol processing of the MCU, the processed data are wirelessly transmitted to the upper computer through Bluetooth.

**Figure 5:**
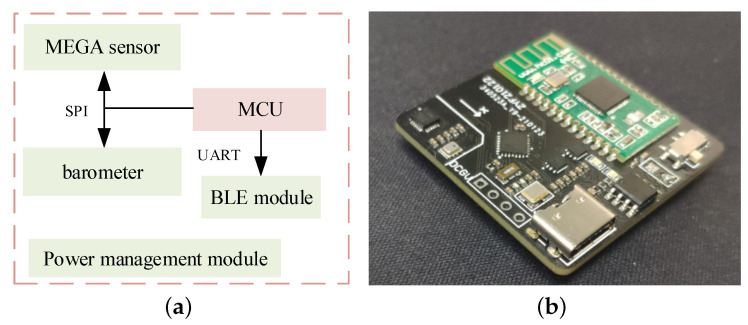
(**a**) System hardware block diagram (**b**) Physical diagram.

The hardware system mainly consists of an MAGR sensor ICM-2098, an air pressure sensor BMP280, a low-power MCU stm32L103, a communication module and a power supply module. The BLE5.0 is used for data communication, with size of 35 mm × 35 mm × 35 mm. The hardware system is powered by a 400 mAh lithium battery, which can be charged through the Type-C interface. On the premise of meeting the selection requirements, the hardware system should use chips with small size and low power consumption as much as possible to increase the working time and reduce the volume. The system hardware block diagram and physical diagram are shown in Figure 5.

### 5.2. Experiment and Analysis

In order to verify the performance of the proposed algorithm, experiments were conducted in the complex building of the Institute of Microelectronics of the Chinese Academy of Sciences. The experimental area was 72.8 m × 32.7 m. The sensors were fixed on the surface of the tester’s shoes, and the collected data were wirelessly transmitted to the host computer in real time through Bluetooth. The sensor data were used to restore the trajectory.

### 5.3. Experiment I: 2D Positioning

Experiment I is mainly to verify the 2D positioning accuracy of different algorithms on the same floor. The algorithms for comparison include ZUPT-based inertial navigation algorithms and cascaded filtering algorithms.

**Figure 6:**
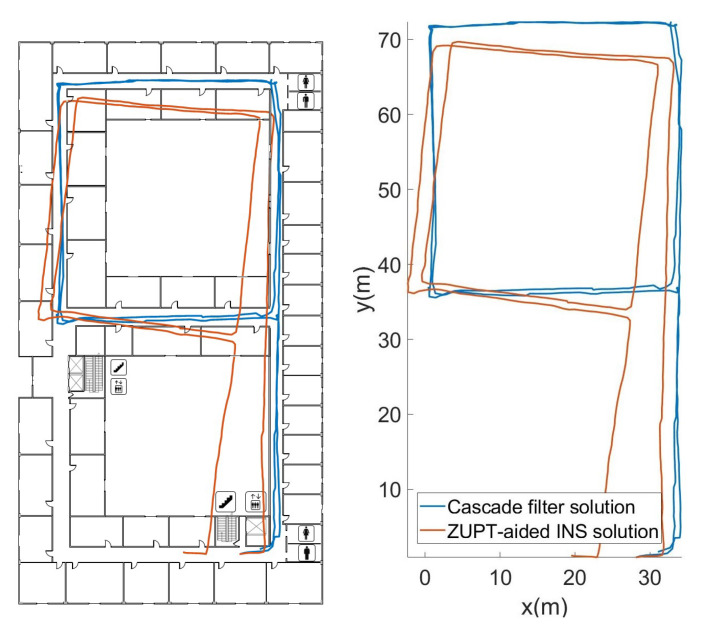
Trajectory restored by different algorithms.

The orange trajectory in Figure 6 was obtained by attitude angle correction based on ECF and zero-velocity-update (ZUPT) correction. Because there is no fixed correction reference for the heading angle direction, the result has a certain accumulated error. The blue trajectory was obtained by the cascade filtering algorithm that combines particle filtering and map information. It can be seen that the cascade filtering algorithm effectively corrects the cumulative error of the heading angle and position in the lower filtering process.

**Figure 7:**
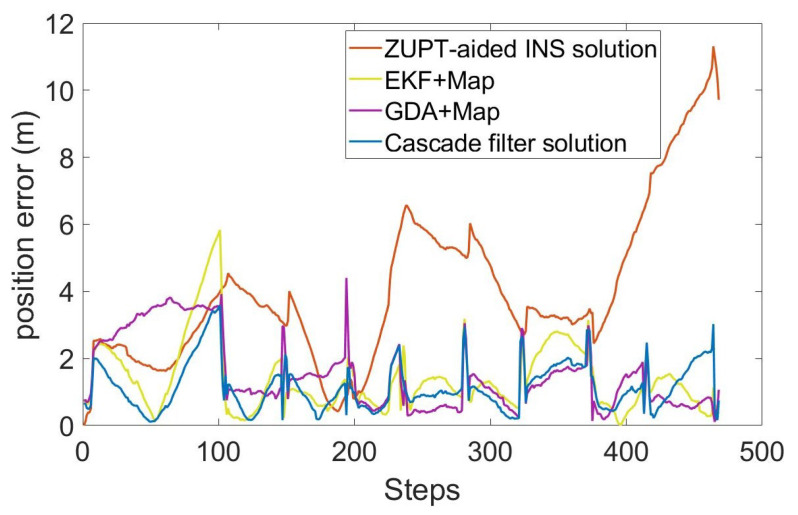
Position error of each step.

**Figure 8:**
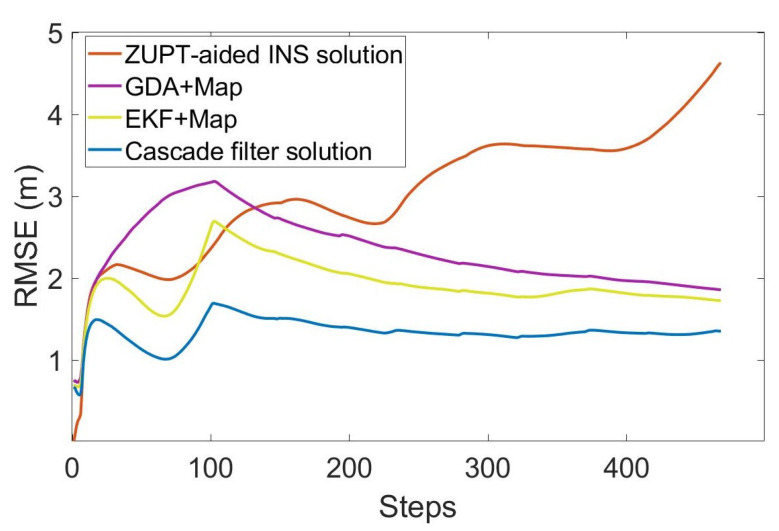
RMS error of each step.

In order to evaluate the performance of the algorithm, we compared the ZUPT-aided INS method that does not contain map information, gradient descent algorithm (GDA) [26], extended Kalman filtering (EKF) [27] and cascade filtering algorithms that integrate the same map information. The position error and RMS error of different algorithms are shown in Figure 7 and Figure 8.

**Figure 9:**
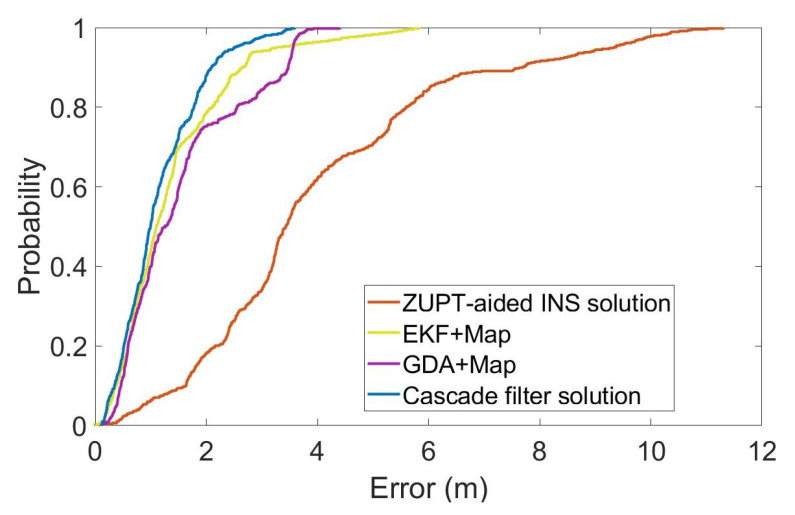
Statistical distribution of position error.

For a more intuitive analysis, we have calculated the cumulative errors of the different methods in the whole process, as shown in Figure 9. The vertical axis is the percentage of the cumulative error, and the horizontal axis is the position error. Table 1 shows the root mean square error (RMSE), average error and maximum position error of each method, calculated according to the cumulative distribution of errors.

The total length of the entire test is 336.6m, including the multiple-turns process. As can be seen in Figure 7 and Figure 8 and Table 1, the RMS error of using inertial navigation alone for the positioning accumulates continuously with the increase in time, while the positioning method combined with the map information has a certain correction effect in the error accumulation. In the case of using the same map information, compared with the GDA and ECF, the cascade filtering algorithm adopted in this paper can preliminarily calibrate the attitude information in the ZUPT process through the ECF algorithm in the lower filtering process. In addition, in the upper filtering process, we complete the correction of the cumulative error of the heading angle by integrating the map information into the particle filter, thereby improving the positioning accuracy.

### 5.4. Experiment II: 3D Positioning

When locating the height using the barometer, there will be some fluctuations due to the uneven indoor air pressure and temperature. When pedestrians walk in a building, the floor changes only when they go up and down through stairs or elevators. Therefore, we marked the positions of the stairs and elevators on the indoor map as a basis for correcting the barometer data by combining the map information. When the barometer changes rapidly and the pedestrian is near the elevator or stairs, it means that the pedestrian is in the state of going up and down the stairs, and the original air pressure data remain unchanged. When a pedestrian walks on the same floor, the barometer does not change drastically. At this time, the floor information can be classified by clustering.

**Figure 10:**
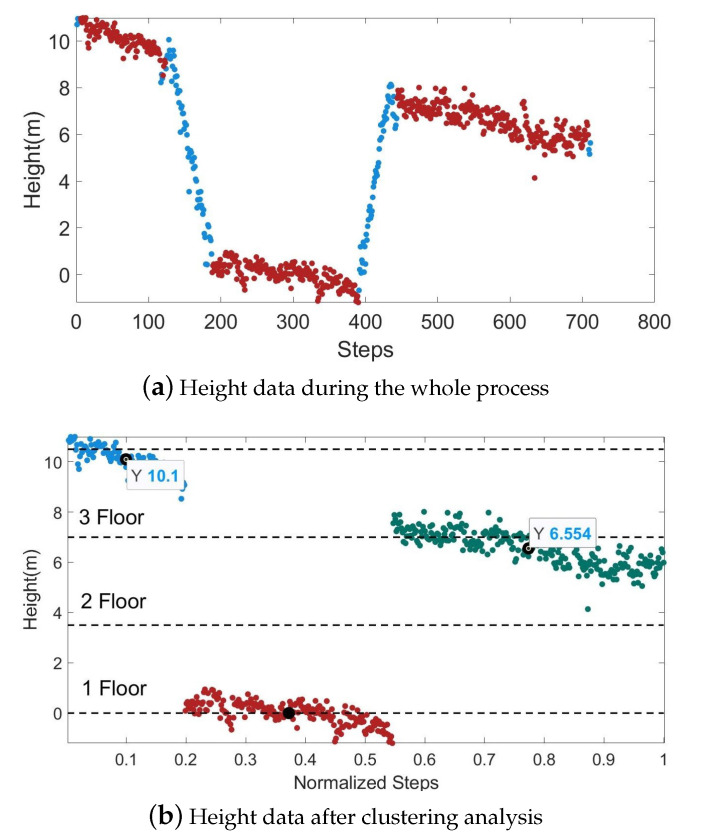
Schematic diagram of the barometer’s height correction process in 3D positioning.

In Figure 10, the horizontal coordinates represent the number of pedestrian steps and the normalized results. The red scatter points represent the barometer data of the pedestrians on a certain floor before the height correction, and the blue scatter points represent the barometer data of the entire 3D positioning process after the correction. Figure 10b shows the result of the clustering analysis on the red scatter in Figure 10a, and the dotted line indicates the real floor height. In this experimental building, the height between adjacent floors is 3.5m. By calculating the mean value of the height data of the same cluster as the measured height of the corresponding floor, we get that the errors of the third and fourth floors are 0.45m and 0.40m. Combining the floor height information in the map, this error level will not cause an incorrect floor identification.

**Figure 11:**
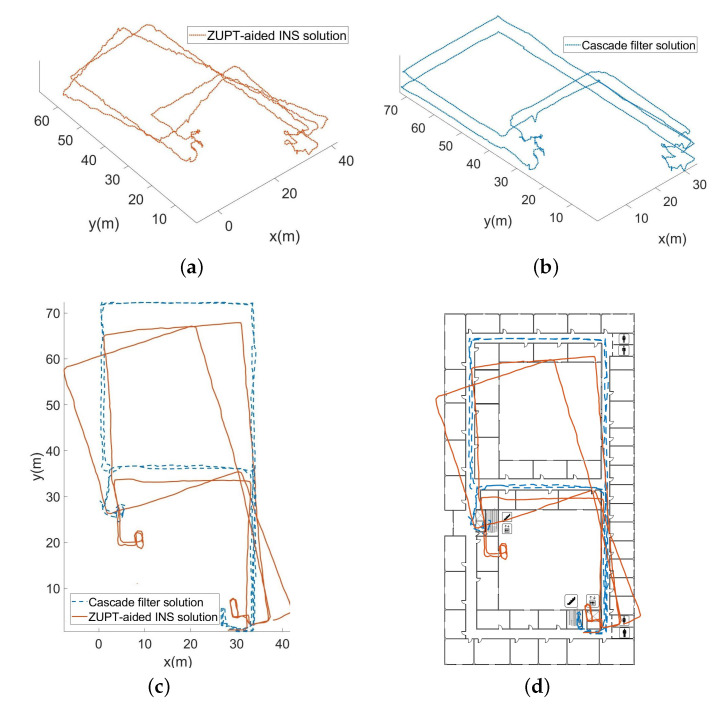
Three-dimensional trajectory restoration and corresponding planar projection. (**a**) 3D trajectory before correction. (**b**) 3D trajectory after correction. (**c**) Plan projection of 3D trajectory. (**d**) Plan projection of 3D trajectory with map information.

Figure 11a shows the 3D trajectory obtained by using the barometer data before correction and the ZUPT-aided inertial navigation algorithm. Figure 11b shows the 3D trajectory obtained by the corrected barometer data and the cascade filtering algorithm. The plane projection of the 3D trajectory restored by the two algorithms is shown in Figure 11c,d.

During experiment II, the whole length is 536.5 m. As the overall measurement time is relatively long and includes complicated processes, such as going up and down stairs, the absolute error at the end position before the correction is 1.88%. However, after the map information constraints and the data correction of the barometer, the absolute error at the end position is reduced to 0.46%, and the 3D positioning accuracy has been effectively improved.

## 6. Conclusions

In this paper, a cascaded filtering algorithm integrating MEMS sensors is used for an indoor location. By combining the particle filter algorithm with the map information, the problem of error accumulation caused by the lack of the correction data of the heading angle in the process of lower filtering is corrected. The experiment shows that the RMS error based on the cascaded filtering algorithm is only 1.35 m in the plane positioning. In addition, we have carried out a cluster analysis according to the data characteristics of the barometer movement on the same floor and the process of going up and down stairs, combined with the floor height information to achieve the height direction correction. In the 3D positioning process with a total length of 536.5 m, the end-point position error is only 0.46%. When judging the pedestrian status, the system only needs a few subsequent prediction steps, which basically meets the real-time positioning. Therefore, it can be used for the long-term, high-precision, real-time positioning in buildings. In addition, the whole process does not require an additional equipment deployment, which can reduce the cost and workload of later maintenance, and it expands the application scenario of this system. In the future, we will further try to apply this system to indoor and outdoor seamless navigation and positioning.

## Figures and Tables

**Table 1:** Error analysis of 2D positioning.

Algorithms	RMSE (m)	Average Error (m)	Maxmum Error (m)
ZUPT-aided INS	4.63	4.01	11.31
GDA+Map	1.86	1.54	4.40
EKF+Map	1.72	1.38	5.84
Cascade filter	1.35	1.15	3.52
